# Mullerian Inhibiting Substance Suppresses Proliferation and Induces Apoptosis and Autophagy in Endometriosis Cells *In Vitro*

**DOI:** 10.1155/2013/361489

**Published:** 2013-06-19

**Authors:** Mostafa A. Borahay, Fangxian Lu, Bulent Ozpolat, Ibrahim Tekedereli, Bilgin Gurates, Sinem Karipcin, Gokhan S. Kilic

**Affiliations:** ^1^Department of Obstetrics & Gynecology, University of Texas Medical Branch, 301 University Boulevard, Galveston, TX 77555-0587, USA; ^2^Division of Cancer Medicine, Department of Experimental Therapeutics, University of Texas MD Anderson Cancer Center, Houston, TX 77030, USA; ^3^Department of Obstetrics & Gynecology, Firat University, 23119 Elazig, Turkey

## Abstract

*Objective*. To determine the effects of Mullerian inhibiting substance (MIS) treatment on endometriosis cells through study of apoptosis and autophagy. *Design*. Experimental *in vitro* study. *Setting*. University research laboratory. *Cell Line*. CRL-7566 endometriosis cell line. This line was established from a benign ovarian cyst taken from a patient with endometriosis. *Interventions*. *In vitro* treatment with MIS. *Main Outcome Measures*. The main outcome measures were cellular viability, proliferation, cell-cycle arrest, and induction of apoptosis and autophagy in endometriotic cells. *Results*. MIS treatment inhibited proliferation of endometriosis cells and induced apoptosis, as indicated by Annexin V staining, and induced caspase-9 cleavage and cell-cycle arrest, as evidenced by increased expression of p27 CDK-inhibitor. MIS treatment also induced autophagy in endometriosis cells as demonstrated by a significant increase in LC3-II induction, a hallmark of autophagy. *Conclusions*. MIS inhibits cell growth and induces autophagy, as well as apoptosis, in ectopic endometrial cell lines. Our results suggest that MIS may have a potential as a novel approach for medical treatment of endometriosis. Further studies may be needed to test the efficacy of MIS treatment in animal models and to develop MIS treatment specifically targeted to the endometriosis.

## 1. Introduction

Endometriosis, one of the most common and debilitating diseases in women, is characterized by endometrial-like tissue outside the uterus. As a major cause of pelvic pain and infertility, it affects 6% to 10% of women of reproductive age, 50% to 60% of women and teenage girls with pelvic pain, and up to 50% of women with infertility [1]. Endometriosis also has a great impact on healthcare system. Annual healthcare costs and costs of productivity loss associated with endometriosis were estimated at $22 billion in 2002 [2].

Current treatments for endometriosis have their shortcomings. Long-term treatment of patients with endometriosis involves repeated courses of medical therapy, surgical therapy, or both. Medical modalities include nonsteroidal anti-inflammatory drugs (NSAIDs), combined contraceptive pills, oral progestogens, depot medroxyprogesterone acetate, danazol, and GnRH agonists. In addition, aromatase inhibitors have been reported to be efficacious in some observational trials, but data are insufficient to recommend their routine use. Mainstays of surgical treatment include laparoscopic ablation of endometriotic lesions, surgical excision of large endometriomas, and unilateral or bilateral adnexectomy. Hysterectomy with bilateral salpingo-oophorectomy, often regarded as definitive therapy for endometriosis, is associated with a 10% chance of recurrent disease [3]. Inefficacy, limitations, side effects, and high incidence of recurrence after current medical treatment options make it imperative to search for new treatment modalities.

Mullerian inhibiting substance (MIS), a 140-kDa homodimer glycoprotein, and a member of TGF-B superfamily of biologic response modifiers cause regression of Mullerian ducts in developing male embryos. The Mullerian duct, which forms the coelomic epithelium, develops into fallopian tubes, uterus, cervix, proximal vagina, and surface epithelium of the ovary in the female [4]. Recent research speculates that tumors derived from Mullerian tissues or expressing MIS type II receptors could respond to MIS in growth inhibition assays [5–7]. In light of MIS causing growth arrest in tumors originating from coelomic epithelium, we hypothesized that ectopic endometrial cells could respond to MIS and undergo growth arrest and programmed cell death. MIS is also known as antimullerian hormone (AMH). For consistency, we will only use the name Mullerian inhibiting substance throughout this work.

## 2. Materials and Methods

### 2.1. Cell Line Culture and Propagation

In these experiments, we used CRL-7566 endometriosis cell line (ATCC, Manassas, VA, USA) [8]. This cell line was established from a benign ovarian cyst taken from a patient with endometriosis. The cells were isolated from both the inner and outer surfaces of the cyst. Cells were grown in Dulbecco's Modified Eagle's Medium (ATCC, Manassas, VA, USA) containing 10% fetal bovine serum (FBS), which was not heat-inactivated. Briefly, the cell layer was rinsed with 0.25% (w/v) Trypsin-0.53 mM EDTA solution to remove all traces of serum containing trypsin inhibitor. Then, 2.0 to 3.0 mL of Trypsin-EDTA solution was added to the dish, and cells were observed under an inverted microscope until the cell layer is dispersed (usually within 5 to 15 minutes). To avoid clumping, cells were not agitated by hitting or shaking the dish while waiting for the cells to detach. Cells that were difficult to detach were brought to 37°C to facilitate dispersal. Then, 6.0 to 8.0 mL of complete growth medium was added, and cells were aspirated by gently pipetting. Finally, appropriate aliquots of the cell suspension were added to new culture vessels. Cells were incubated in an atmosphere of 95% air and 5% carbon dioxide (CO_2_) at a temperature of 37°C. Culture medium was changed every 2 to 3 days.

### 2.2. Mullerian Inhibiting Substance

We used recombinant human Mullerian inhibiting substance (MIS) from R&D Systems (Minneapolis, MN, USA). Per vendor recommendation, MIS was reconstituted at a concentration of 10 *μ*g/mL in sterile phosphate buffered saline (PBS) containing 0.1% human serum albumin.

### 2.3. Cell Viability and Growth Assays

Cultured CRL-7566 cells were harvested at 80% confluence and placed in 96-well plates at 2000 cells per well in 100 *μ*L of media per well. After 24 hours of incubation, we treated cells with recombinant human MIS at 2 different concentrations: 10 *μ*g/mL and 5 *μ*g/mL. Additional wells were also treated with PBS (pH = 7.4) as a buffer control. After 4 days of incubation, the medium was changed by adding MIS and buffer control to respective wells, and plates were shaken. All experiments were performed in duplicate.

To evaluate cell viability, MTT (3-(4,5-dimethylthiazol-2-yl)-2,5-diphenyltetrazolium bromide) cell proliferation assay (R&D Systems, Minneapolis, MN, USA) was performed on the seventh day of treatment. The MTT reagent was added (10 *μ*L per well), and the plates were incubated for 3 hours to allow for intracellular reduction of the soluble yellow MTT to the insoluble purple formazan dye. Cells were viewed periodically for the appearance of punctate, intracellular precipitate, using an inverted microscope. Subsequently, the medium was gently replaced by 100 *μ*L of dimethyl sulfoxide (DMSO) per well, including control wells, to solubilize the formazan dye. Plates were left covered in the dark at 18°C–24°C for 2 hours with shaking. Plates were read at 595 nm wavelengths in an Elisa plate reader (Kinetic Microplate Reader, Molecular Devices Corporation, Sunnyvale, CA, USA). Absorbance was proportional to the relative abundance of viable cells in any given well. MIS-treated and untreated samples were compared. All experiments were performed in duplicate.

### 2.4. Propidium Iodide Staining for Cell-Cycle Analysis

The cells were plated into 6-well plates at 1 × 10^6^ cells/well and treated with MIS for 7 days. The control and treated cells were harvested with trypsin-EDTA and washed in 1 mL of cold PBS. The cells were resuspended in 0.5 mL of propidium iodide solution (50 *μ*g/mL propidium iodide, 0.1% Triton X-100, and 0.1% sodium citrate in water). Cells were incubated at 4°C for 2 hours in the dark, and fluorescence was then read on a Coulter Epics XL flow cytometer (Beckman Coulter, Brea, CA, USA). Cells in sub-G1 phase were quantified and are presented as the percentage of the total cells.

### 2.5. Analysis of Apoptosis

Apoptosis was assessed by Annexin V staining, and positive cells were quantified by flow cytometry. To provide a comparative assay of apoptosis by Annexin V labeling, endometriosis cells (1 × 10^6^) treated with MIS for 24–96 hours were harvested and washed with PBS. Cells were resuspended in a binding buffer and stained with Annexin V and propidium iodide (PI) according to the manufacturer's protocol (BD Pharmingen Annexin V kit). Positive cells were detected and quantified by FACS analysis.

### 2.6. Flow-Cytometric Analysis of Apoptosis

One of the earliest changes of apoptosis is that the membrane phospholipid PS translocates from the inner to the outer leaflet of the membrane. Thus, PS is exposed to the external membrane and can be detected using PS-binding protein such as Annexin V. To provide a comparative assay of apoptosis by Annexin V labeling, tumor cells (1 × 10^6^) treated with MIS or left untreated as control for 4 days were harvested, washed, fixed with ice-cold 70% ethanol (50 min, 4°C), and resuspended in binding buffer (10 mM HEPES/NaOH (pH 7.4), 140 mM NaCl, and 2.5 mM CaCl_2_). Fifty *μ*L of FITC-Annexin V (R&D Systems, Minneapolis, MN, USA) was added and incubated for 15 minutes in the dark at room temperature before flow-cytometric analysis. 

### 2.7. Western Blot Analysis

After treatment, cells were collected and centrifuged, and whole-cell lysates were obtained using a lysis buffer. Total protein concentration was determined using a DC protein assay kit (Bio-Rad, Hercules, CA, USA). Aliquots containing 30 *μ*g of total protein from each sample were subjected to sodium dodecyl sulfate-polyacrylamide gel electrophoresis (SDS-PAGE) with a 4%–20% gradient and electrotransferred to nitrocellulose membranes. The membranes were blocked with 5% dry milk in tris-buffered saline-tween 20 (TBST), probed with primary antibodies diluted in TBST containing 2.5% dry milk, and incubated at 4°C overnight. We used primary antibodies against cleared caspase-9, p27, p-ERK1/2 (MAPK), Beclin-1, and LC-3 from Cell Signaling Technology (Beverly, MA, USA). After being washed, the membranes were incubated with horseradish peroxidase-conjugated anti-rabbit or anti-mouse secondary antibody (Amersham Life Science, Cleveland, OH, USA). Mouse anti-*β*-actin and donkey anti-mouse secondary antibody were purchased from Sigma Chemical (St. Louis, MO, USA) so that *β*-actin expression could be monitored to ensure equal loading of proteins. Chemiluminescent detection was performed with ChemiGlow (Alpha Innotech, San Leandro, CA, USA) detection reagents. The blots were visualized with a FluorChem 8900 imager and quantified by a densitometer using the Alpha Imager application program (Alpha Innotech). All experiments were independently repeated at least twice.

### 2.8. Statistical Analysis

The data were expressed as the mean ± SD of 3 or more independent experiments, and statistical analysis was performed using the 2-tailed and paired *t-*test. *P* values less than 0.05 were considered statistically significant. 

## 3. Results

### 3.1. MIS Treatment Inhibits Proliferation of Ectopic Endometrial Cell Line

**Figure 1:**
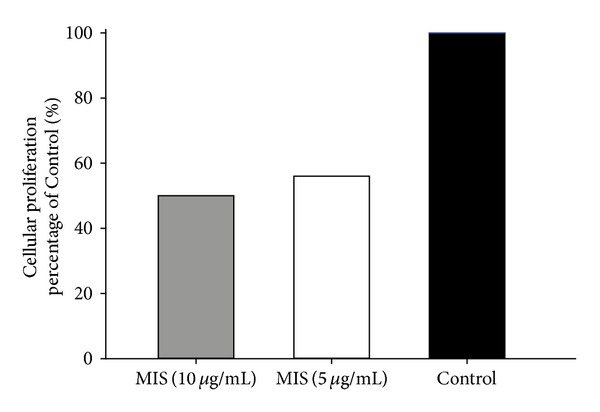
MIS inhibits proliferation of endometriosis cells. Cells of CRL-7566 endometriosis cell line were treated with MIS at concentrations of 10 *μ*g/mL and 5 *μ*g/mL, and cell viability was determined by MTT assay.

We first investigated whether MIS treatment leads to growth inhibition and reduces cell viability in endometrial cells by performing a proliferation assay. MIS caused dose-dependent inhibition in growth of endometriosis cell lines. Maximum inhibition of 50% was observed at 10 *μ*g/mL MIS concentration followed by 44% growth inhibition at 5 *μ*g/mL (Figure 1). 

### 3.2. MIS Treatment Induces p27 in Ectopic Endometrial Cell Line

**Figure 2:**
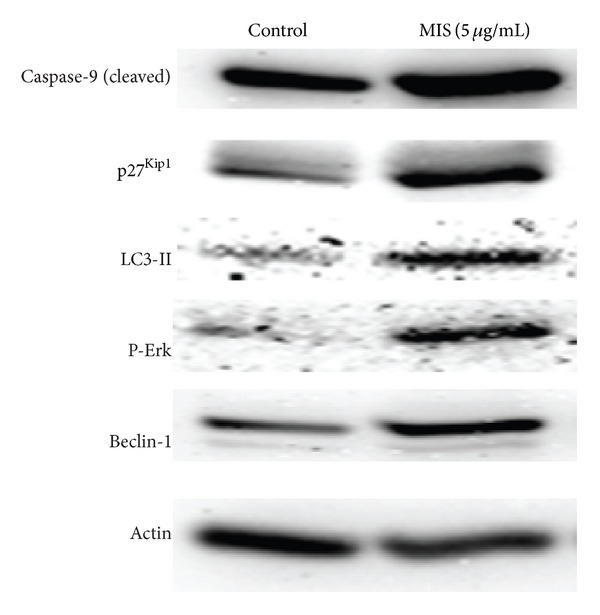
MIS treatment induces apoptosis and autophagy in endometriosis cells. Cells were lysed after MIS treatment at a concentration of 5 *μ*g/mL, and expression of proteins was detected by Western blot analysis using specific antibodies against each protein. *β*-Actin was used as a loading control. Refer to text for further details.

p27^Kip1^ is an inhibitor of cyclin-dependent kinase (CDK) involved in the regulation of the cell cycle. Previous studies showed that expression is significantly decreased in cancers, including endometrial carcinoma [9]. p27 expression is necessary to control the proliferation of endometrium, and its loss of expression seems to play a role in some aspects of endometrial carcinogenesis. Therefore, we sought to determine the expression of p27 after MIS treatment. We found that MIS at a concentration of 5 *μ*cg/mL significantly induced p27 expression in endometrial cell lines, suggesting that MIS may induce growth inhibition through inhibition of cell-cycle suppressor p27 (Figure 2).

### 3.3. MIS Treatment Induces Apoptosis in Ectopic Endometrial Cell Line

**Figure 3:**
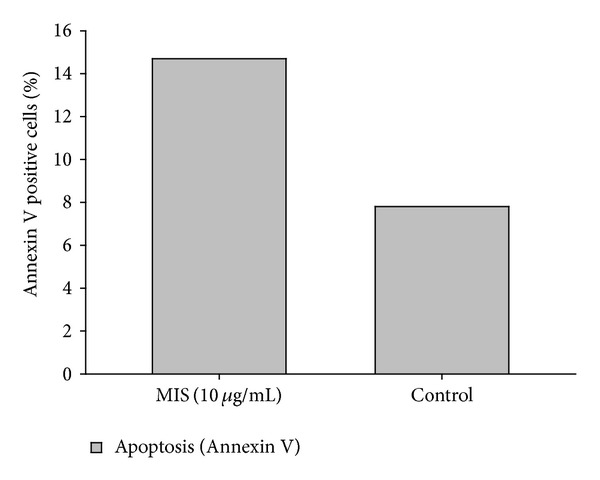
MIS induces apoptosis endometriosis cells. Cells were treated with MIS at a concentration of 10 *μ*g/mL for 96 hours. Apoptotic cells were detected by Annexin V staining and quantified by FACS analysis.

We next investigated the mechanism of growth inhibition induced by MIS by examining cell viability and programmed cell death. Treatment of the cells by MIS caused apoptosis as detected by Annexin V staining (Figure 3). Cell lines treated with MIS at a 10 *μ*g/mL concentration showed 14.7% Annexin positivity, as opposed to the control group with 7.8% positivity.

We also found that MIS treatment induces caspase 9 cleave in Western blots, providing another line of evidence that MIS treatment induces apoptosis in endometriosis cells (Figure 2).

### 3.4. MIS Treatment Induces Autophagy in Ectopic Endometrial Cell Line

Recent studies demonstrated that cells can undergo autophagic cell death, which is a new type of cell death and considered to be programmed cell death (PCD) type II [10, 11]. In addition to apoptosis (PCD type I), autophagic cell death can lead to elimination of normal or cancer cells. To elucidate the mechanism of cell death and growth inhibition, we next determined the effect of the MIS on induction of autophagy by performing acridine orange staining, indicating the formation of the acidic vesicular organelles, followed by FACS analysis for quantitation and Western blot analysis to assess microtubule-associated protein 1 light chain 3-II (LC3-II) expression. During autophagy, LC3, the homologue of the yeast Apg 8/Aut7p gene, is cleaved to LC3-I and conjugated to phosphatidylethanolamine to form LC3-II, a hallmark of autophagy, which is localized on the autophagosomal membrane. The processing of LC3-I to LC3-II is essential for the formation of the autophagosome, and LC3-II levels are proportional to the autophagic vacuole accumulation of LC3-II protein. Treatment of the cells with MIS for 72 hours demonstrated a significant increase in LC3-II induction, a hallmark of autophagy. All of these findings support autophagy induction (Figure 2). Untreated cells under optimal conditions failed to increase LC3-II expression (Figure 2) during the course of therapy; however, treatment of cells with MIS significantly induced LC3-II expression, suggesting that MIS treatment induces autophagy in endometriosis cells. 

### 3.5. MIS Treatment Induces Autophagy by Inducing Beclin-1 and ERK Activity

Next, we determined the mechanism by which MIS treatment induces autophagy in endometriosis cells. Beclin-1, the product of autophagy promoting gene Beclin-1 (Atg6), has been shown to regulate autophagy in cancer cells. Therefore, we sought to determine whether MIS induces autophagy by induction of Beclin-1. We found that MIS treatment induces Beclin-1 autophagy-promoting protein in cells (Figure 2). Untreated control cells failed to induce Beclin-1 and autophagy. These findings suggested that MIS treatment induces autophagy through induction of Beclin-1 in endometriosis cells. 

Because increased activation (phosphorylation) of ERK has been linked to induction of autophagy, we also investigated if ERK activity is induced. We found that phosphorylation of ERK and expression of Beclin-1 are induced after MIS treatment, suggesting that MIS may induce autophagy through ERK and Beclin-1 autophagy-promoting protein (Figure 2).

## 4. Discussion

Early development of the reproductive system is similar in both sexes in that gonads and Mullerian ducts are comparable. Subsequent gender differentiation starts when, in females, the Mullerian system develops into the upper vagina, cervix, uterus, and fallopian tubes, and, in males, the Mullerian duct degenerates under the effect of Mullerian-inhibiting substance [12]. 

MIS, first described by Jost in 1947 [13], is a 140-kDa homodimeric glycoprotein member of the transforming growth factor-beta (TGF-*β*) superfamily. In embryos, it is secreted by Sertoli cells in the embryonic testes, whereas it is absent in female embryos. In males, MIS levels are elevated throughout infancy and childhood, slowly decreasing just before puberty when a fall in MIS precedes the onset of puberty. The lower levels are subsequently maintained throughout male adult life in the 2–5 ng/mL range. In females, conversely, MIS serum levels are undetectable until the prepubertal period, when serum MIS levels increase to the adult range (2–5 ng/mL) and are maintained until menopause when MIS is no longer detectable [14–16].

The exact biological functions of MIS in adult females are uncertain; however, MIS is most likely involved in regulation of initial primordial follicle recruitment and selection [17, 18]. Currently, MIS is used as a sensitive marker, especially in the process of *in vitro* fertilization, to assess the ovarian reserve. 

A growing body of evidence supports the idea that MIS functions as a regulator of cell growth and can be used to inhibit the growth of cells and tissues of Mullerian and coelomic epithelial origins. For example, MIS has been shown to inhibit the growth of ovarian, cervical, and breast neoplasms [19–24]. These findings suggest a potential role for MIS in postembryonic regulation of growth. Renaud et al. [5] showed that both normal human endometrium and endometrial cancers express the receptor for MIS and that MIS can inhibit the proliferation of a number of human endometrial cancer cell lines expressing the MIS type II receptor. In their research, they showed that MIS affects the expression of key cell-cycle regulatory proteins in the representative endometrial cancer cell line AN3CA [5]. 

In this study, we evaluated the effect of *in vitro* treatment of endometriosis cells with MIS. Our results showed that MIS treatment inhibits proliferation and induces apoptotic cell death in ectopic endometrial cells. We also revealed that MIS treatment induces autophagy in endometriosis cells. We and others have shown that induction of autophagy leads to cell death [10, 11, 25, 26]. We also found that MIS induces autophagy through increased expression of Beclin-1 and activation (phosphorylation) of ERK, both of which have been shown to induce autophagy. This is the first study to look at the apoptosis and autophagy at the same time. The autophagy pathway warrants exploration in future studies to gain a better understanding of additional mechanisms of cellular death, revealing the association between autophagy and apoptosis. 

MIS appears to work in endometrial tissues mainly through an autocrine/paracrine mechanism [27]. This is consistent with experiments showing that the MIS secreted by each testis induces regression of only the ipsilateral Mullerian duct [28]. This is also consistent with the finding that serum levels of MIS are significantly lower than the levels that inhibit cellular growth and induce apoptosis [27].

Recently, it was demonstrated that infertile patients with minimal and mild endometriosis had lower serum MIS levels compared to a cohort of infertility patients with tubal occlusion but no endometriosis [29]. However, serum MIS levels do not show a significant fluctuation during a spontaneous menstrual cycle. We can conclude that the effect of this hormone is mainly restricted to the ovarian follicular milieu, and its mechanism needs to be clarified. It is more likely that low serum MIS levels in patients with mild or moderate endometriosis reflect a low ovarian reserve rather than being a cause or result of endometriosis itself. 

In conclusion, this study suggests that MIS has the potential to inhibit proliferation and induce regression in ectopic endometrial tissues. Further studies are needed to evaluate MIS as a potential novel therapeutic approach to avoid surgical intervention. This includes studying *in vivo* effect of MIS on endometriosis animal models. Furthermore, additional work is needed to specifically target ectopic endometrial tissue with MIS to avoid possible effects on other coelomic epithelium-originated tissues.
